# The Extent of User Involvement in the Design of Self-tracking Technology for Bipolar Disorder: Literature Review

**DOI:** 10.2196/27991

**Published:** 2021-12-20

**Authors:** Shazmin Majid, Stuart Reeves, Grazziela Figueredo, Susan Brown, Alexandra Lang, Matthew Moore, Richard Morriss

**Affiliations:** 1 School of Computer Science Horizon Centre for Doctoral Training University of Nottingham Nottingham United Kingdom; 2 School of Computer Science University of Nottingham Nottingham United Kingdom; 3 National Institute for Health Research MindTech Medtech Co-operative Institute of Mental Health University of Nottingham Nottingham United Kingdom; 4 Human Factors Research Group Faculty of Engineering University of Nottingham Nottingham United Kingdom; 5 Institute of Mental Health University of Nottingham Nottingham United Kingdom; 6 National Institute for Health Research Applied Research Collaboration East Midlands Institute of Mental Health University of Nottingham Nottingham United Kingdom; 7 Nottingham National Institute for Health Research Biomedical Research Centre Institute of Mental Health University of Nottingham Nottingham United Kingdom

**Keywords:** user-centered design, participatory design, human-computer interaction, patient and public involvement, self-monitoring technology, bipolar disorder, mobile phone

## Abstract

**Background:**

The number of self-monitoring apps for bipolar disorder (BD) is increasing. The involvement of users in human-computer interaction (HCI) research has a long history and is becoming a core concern for designers working in this space. The application of models of involvement, such as user-centered design, is becoming standardized to optimize the reach, adoption, and sustained use of this type of technology.

**Objective:**

This paper aims to examine the current ways in which users are involved in the design and evaluation of self-monitoring apps for BD by investigating 3 specific questions: are users involved in the design and evaluation of technology? If so, how does this happen? And what are the best practice *ingredients* regarding the design of mental health technology?

**Methods:**

We reviewed the available literature on self-tracking technology for BD and make an overall assessment of the level of user involvement in design. The findings were reviewed by an expert panel, including an individual with lived experience of BD, to form best practice *ingredients* for the design of mental health technology. This combines the existing practices of patient and public involvement and HCI to evolve from the generic guidelines of user-centered design and to those that are tailored toward mental health technology.

**Results:**

For the first question, it was found that out of the 11 novel smartphone apps included in this review, 4 (36%) self-monitoring apps were classified as having no mention of user involvement in design, 1 (9%) self-monitoring app was classified as having low user involvement, 4 (36%) self-monitoring apps were classified as having medium user involvement, and 2 (18%) self-monitoring apps were classified as having high user involvement. For the second question, it was found that despite the presence of extant approaches for the involvement of the user in the process of design and evaluation, there is large variability in whether the user is involved, how they are involved, and to what extent there is a reported emphasis on the voice of the user, which is the ultimate aim of such design approaches. For the third question, it is recommended that users are involved in all stages of design with the ultimate goal of empowering and creating empathy for the user.

**Conclusions:**

Users should be involved early in the design process, and this should not just be limited to the design itself, but also to associated research ensuring end-to-end involvement. Communities in health care–based design and HCI design need to work together to increase awareness of the different methods available and to encourage the use and mixing of the methods as well as establish better mechanisms to reach the target user group. Future research using systematic literature search methods should explore this further.

## Introduction

### Overview

**Figure 1:**
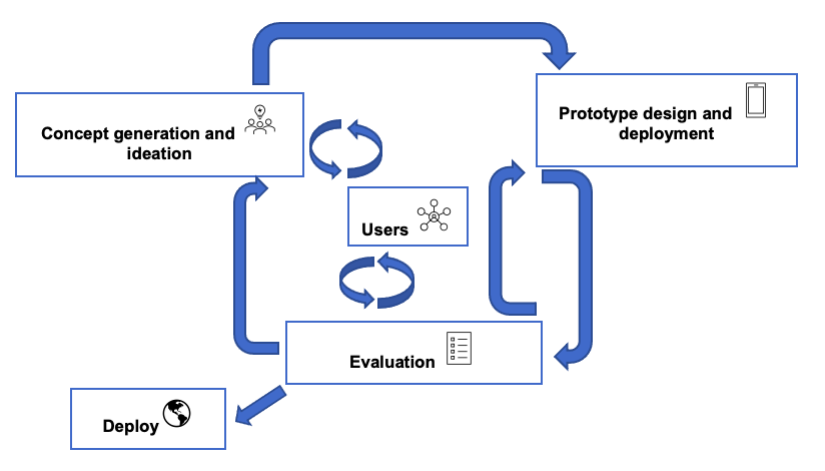
User-centered design process adapted from McCurdie et al [5].

Smartphone apps focused on mental health are increasing in number [1]. There are approximately 10,000 mental health and wellness apps available for download for mental health diagnosis, treatment, and support. Self-monitoring apps are predominant, and it was found that most applications for serious mental illnesses (such as bipolar disorder [BD], schizophrenia and schizoaffective disorder, major depressive disorder, and psychotic disorder with suicidality) fall into this category [2]. As a case example, we focus on self-monitoring apps for BD. Despite the growth of this market, little supporting literature exists to guide best practice design and evaluation of the effectiveness of mental health apps [3]. Murray et al [4] have argued that to establish and optimize the reach, adoption, and sustained use of health interventions, the principles of user-centered design (UCD) are required. The application of participatory approaches such as UCD principles and activities (International Organization for Standardization 9241-210:2010) are becoming standardized more increasingly, an example being the approaches used by the National Health Service in the United Kingdom and also internationally in private and public health and the industries that provide them, such as medical technology and pharmacological companies. Given that UCD is a commonly adopted approach, we first need to explain how it is articulated to contextualize this to other approaches of user involvement that are being adopted. The principle is outlined as follows and further illustrated in Figure 1 [5]:

1. The design is based on an implicit and explicit understanding of users, tasks, environments, and interactions in the context.
2. Users are involved throughout design and development.
3. The design is driven and refined by user-centered evaluation.
4. The process is iterative.
5. The design addresses the whole user experience.
6. The design team includes multidisciplinary skills and perspectives.

This paper presents the current landscape of user involvement approaches in the design and evaluation of self-monitoring apps for BD. This is investigated via 3 specific questions: (1) are users involved in the design and evaluation of technology? (2) if so, how does this happen? and (3) what are the best practice *ingredients* regarding the design of mental health technology?

Regarding the first question, Goodwin et al [6] state that there is a lack of parity of user involvement in the design of physical and mental health apps, where for mental health, users are involved less frequently than they are for physical health. A recent review [7] examined the last decade of studies on affective health (including BD) and human-computer interaction (HCI). User involvement was considered, and it was identified that more ethically sensitive design practices, including the voices of people living with affective disorders, need to be integrated. Of the 139 publications included in this study, only 16 (11.5%) of the studies reviewed reported *clinical evaluations* described as involving service users of mental health services or which met the formal criteria for a specific mental health problem. This lack of user involvement is reflected in the quality of mental health apps for BD as Nicholas et al [8] established that a significant proportion of apps contained wish list requests, indicating that users’ needs are not being met by current app designs.

Understanding how a serious mental health condition such as BD has an impact on daily experiences is important and helpful when designing technology to create a meaningful technological experience. A study that examined the pathology of BD [9] described the following unique design considerations for mobile technology that have been reported in the literature: (1) the side effects of medication (such as lithium) on a user’s ability to read on-screen text, (2) the impact of medication nonadherence in BD and how this may affect engagement, (3) sensitivity to reward-based stimuli in BD and how this can be used for novel interventions, and (4) the association of increased creativity in BD and the suggestion that such a user group could contribute greatly compared with other users in involvement-orientated type design.

**Figure 2:**
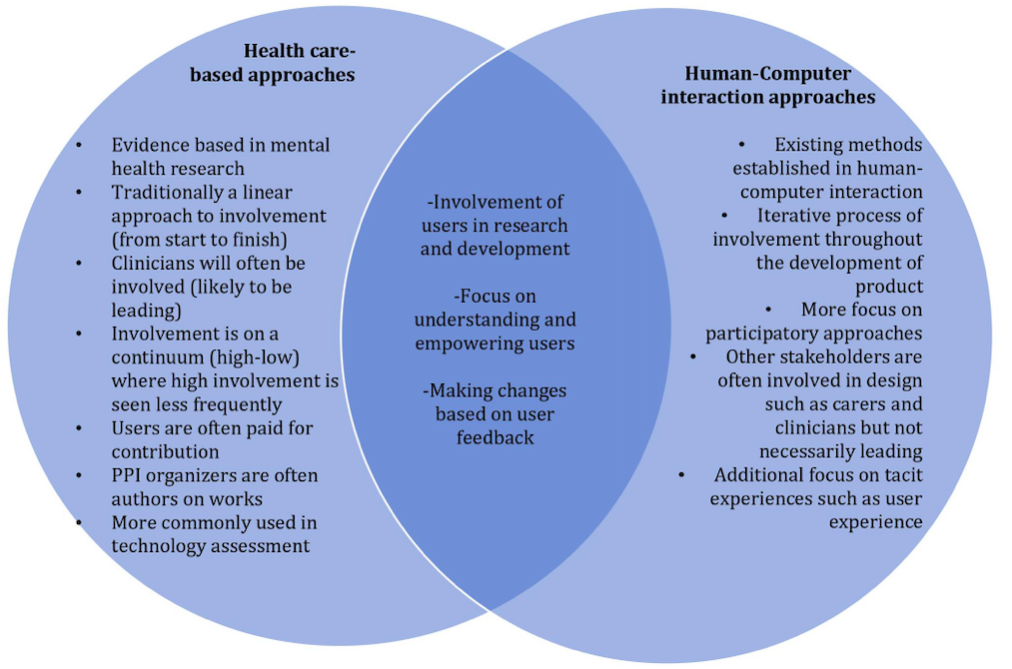
Applications, overlaps, and tensions of health care–based design approaches and human-computer interaction approaches. PPI: patient and public involvement.

Regarding the second question, it is important to understand that user involvement in the design of such technology involves an intersection between health care and technology development, both of which traditionally have different approaches when involving the user, although there are exceptions to the rule. Patient and public involvement (PPI) dominates as concept for *involvement and engagement* within health care studies [10] and improvement. UCD dominates within HCI or service design more broadly and integrates participatory approaches to achieve the involvement and engagement of end users. Both of these approaches look to provide a voice to the patient end users within applied development projects. It is important to consider these approaches and how they are applied, paying particular attention to where they overlap and where the tensions lie, some of which are outlined in Figure 2.

### Current Research

There is a growing body of research on the design of mental health technology with user input, but little has been done to compare approaches of user involvement to understand best practice. Therefore, this paper explores the current practices of user involvement in the design of mental health technology by surveying the available literature, using self-tracking technology for BD as a case example. Specifically, we aim to understand what practices are being used and the extent to which they involve the user. We acknowledge that there can be multiple stakeholders, participants in and *users* of mental health technology but as self-reflection and awareness are the main aim of self-tracking systems, in this instance we define *users* as those who have a diagnosis of BD who are the primary users of this technology. To explore these practices, we review the available literature on self-tracking technology for BD and make an overall assessment of the level of user involvement in design. The findings from this review are used to form best practice *ingredients* for the design of mental health technology. This combines the existing practices of PPI and HCI to evolve from the generic guidelines of UCD and to those tailored toward mental health technology.

### Background

#### Health Care–Based Design Approaches

PPI is described as the involvement of patients, carers, and the public as *active partners* in the design, delivery, and dissemination of research to ensure its relevance and usefulness. In other words, *research is carried out with or by members of the public rather than to, about or for them* [11]. Unlike the design of mental health technology, guidelines exist on the best practices for PPI and measuring its effectiveness. Regarding practices, there is a continuum of PPI, which is closely linked to Arnstein Ladder of Citizen Engagement [12]. Involvement occurs at different levels, and each level has a corresponding level of effort, commitment, and potential impact or outcome. This ladder stretches from tokenism to being fully embedded, where patients are the more dominant voice, delivering and managing the research themselves. The lower ends of this ladder consist of researchers asking for users’ views, which are then used to refine key study documents such as recruitment materials or to inform research decision-making but does not go so far as to involve the PPI representatives as partners in research activities. Aiming to establish an equal relationship between the researchers and PPI participants with shared decision-making (often referred to as *coproduction*) is at the high end of this participation ladder and described as the pinnacle by many researchers and clinicians, if not PPI participants themselves. In line with best practice, PPI participants should be remunerated for time and effort on projects, although this is variable depending on the research and stage of the project [12]. PPI organizers often PPI participants themselves can also be listed as coauthors in published works. In terms of effectiveness, a systematic review of PPI in health and social research identified the following as benefits of PPI involvement: enhanced quality and appropriateness of research, development of user-focused research objectives, user-relevant research questions, user-friendly information, questionnaires and interview schedules, appropriate recruitment strategies for studies, consumer-focused interpretation of data, and enhanced implementation and dissemination of study results [10].

PPI processes are often used in health technology assessment studies [13], where the most commonly used approach involves patients and members of the public directly participating in committees on the agency involved in technology assessment, which involves the systematic evaluation of the properties, effects, and impacts of health technology. PPI is also being increasingly used in mental health technology development in recent years [14,15] with some more citizen focused approaches. PPI is a more passive exercise held at timed points in the research rather than continuing involvement with direct interaction with technology on an ongoing basis when compared with more technology-based approaches in HCI, which are more iterative and cyclical.

#### HCI Design Approaches

Technology-based approaches in the discipline of HCI have developed a *powerful vocabular*y [16] when it comes to involving the user in design evolving from UCD to more participatory democratic forms, considerations of nonuse, design fictions, critical engagements, and various other approaches. In particular, Orlowski et al [17] touched on 2 specific methods when designing mental health technologies with users, which were utilized in 2 case studies. First is participatory design [18], a Scandinavian-born practice which is characterized by a 3-stage iterative design process aimed at understanding users’ implicit knowledge: (1) exploration of work, (2) discovery processes, and (3) prototyping. Each stage is organized and carried out with the users. Another method mentioned by Orlowski et al [19] stemming from UCD is design thinking. Developing empathy for users is at the heart of design thinking as well as working in collaborative multidisciplinary teams and using *action-orientated rapid prototyping* of solutions. Similar to participatory design, design thinking is an iterative process that includes several rounds of *need-finding*, *ideation* and *implementation*. The interesting part of this cycle is the *need-finding*, which is focused on developing empathy for the users and asking questions, such as *who are we trying to help and what are the social, political, and economic contexts?* When comparing these approaches in HCI with health care–based approaches, such as PPI, participatory design is often referred to as *co-design*, such as medium-level involvement as described in PPI. Design thinking takes this 1 step further by focusing on the development of empathy and achieving parity in voice, which is often described as being absent in PPI. Both approaches are also iterative, which is needed in the development of technology, rather than the linear process in PPI. Moreover, with technology-based approaches, there is already acknowledgment of the various tensions, such as cost and regulation, and how to embed these into the process of development which are detailed in relevant regulations (International Organization for Standardization 9241-210:2010 and International Organization for Standardization/International Electrotechnical Commission 62366).

## Methods

### Study Design

#### Search Strategy

In this review, we used 2 search methods. The papers included were from the hits of searches from an ongoing systematic review that lead author SM was working on, which focused on user preferences for self-monitoring technologies for central nervous system disorders, including BD as a central nervous system disease. This systematic review is registered on the international prospective register of systematic reviews (PROSPERO 2019; CRD42019139319) and used the following search terms: (ehealth OR mhealth OR digital health OR telehealth OR telemonitoring) AND (remote patient monitoring OR remote monitoring technology OR application OR wearable OR app OR device) AND (central nervous system OR psychiat* OR neurolog* OR neurodegen* OR mental health OR chronic) AND (prefer* OR evaluation OR feedback OR usability OR design OR visual*) AND patient AND (data or symptom OR UI OR user interface) AND (disease OR disorder OR condition) on the following databases: Association of Computing Machinery, PubMed, Embase, IEEE Xplore and Web of Science, and the Cochrane Library for papers published in the English language between 2007 and 2019. During abstract search, papers related to BD and self-monitoring technology form part of this review. A further check of the literature was performed on Google Scholar to review missing papers using the following search for papers published in the English language between 2007 and 2019: (bipolar AND app*).

#### Inclusion Criteria

The following inclusion criteria were used for included papers: sample of users with BD and feature novel self-monitoring technology.

### Information Extraction

Papers were screened, and information was extracted by lead author SM. The following information was extracted and forms part of the results: number of participants with BD, description of self-monitoring technology, description of user involvement methods, model of user involvement, and further description of the model of user involvement. This information was assessed according to the criteria of involvement, the results of which are also outlined in the table.

### Assessment Criteria for User Involvement

The assessment criteria for user involvement are based on the best practice model of user involvement, which describes that users should be involved in the concept generation and ideation stage, prototype design and deployment stage, and evaluation stage with mechanisms for iteration as described earlier in the paper (Figure 1). To embed PPI processes, we described the empowerment of decision-making and creating empathy as part of high user involvement, as this was described as high on the PPI continuum of involvement [12]. The criteria are further detailed in Textbox 1.

**Criteria for assessing user involvement in selected papers. The categories used here have been developed specifically for this paper and are different from the degrees of involvement in designing and running a research study as used in patient and public involvement practices.**

**No user involvement mentioned**

- No mention of user involvement in design and evaluation

**Low user involvement**

- Users were only involved in 1 stage of design and evaluation with or without iteration

**Medium user involvement**

- Users were involved in more than one stage of design and evaluation with iteration

**High user involvement**

- Users were involved in the concept generation and ideation stage, prototype design and deployment stage, and evaluation stage with iteration, likely to have explicit mention of empowering decision-making and creating empathy

### Expert Panel Review

The expert panel consisted of an individual with >7 years of lived experience of BD and expertise in PPI, Professor of Psychiatry, Assistant Professor of HCI, Assistant Professor in Human Factors, Research Fellow in Involvement and Implementation, Senior Research Data Scientist and lead author SM, who is a PhD student in HCI with a background in mental health research. Included papers and criteria of assessment were subject to discussion with the expert panel via bimonthly web-based meetings with lead author SM over a period of a year. As the inclusion criteria for the study were only 2-fold, there were no disagreements over the included papers. There were also no disagreements over the criteria of involvement as the papers fell distinctly into these based on extracted information, which is limited by what was reported in the studies. Information extracted from the papers was reviewed, and findings were discussed at meetings to make inferences over what constitutes best practice in this context. The expert panel review involved discussing recommendations as a response to the findings of this study to move toward better practice of user involvement. The findings of the study were used as the basis of discussion, which was further built upon with the expertise and working experience of the panel members of user involvement in mental health technology design. SM led the write-up on the findings and research paper, which was shared with the team for comments and changes that were implemented accordingly.

## Results

### Overview

The results from the literature search are presented in Tables 1 and 2, including the number of participants, description of remote monitoring technology, assessment criteria for user involvement, further description of methods used, and model of user involvement. In total, 4 studies fell into the category of no user involvement mentioned, 3 fell into the category of low user involvement, 4 fell into the category of medium user involvement, and 2 in high user involvement.

**Table 1:** Summary of review including characteristics and reference, number of participants, bipolar disorder-specific, description of self-monitoring technology, assessment criteria for user involvement.

Characteristics and reference			Number of participants, n		Bipolar disorder–specific		Description of self-monitoring technology		Assessment criteria for user involvement
**No user involvement mentioned (n=4)**									
	[20]	22		Yes		Combination of True Colours Monitoring system and customized app that records geographic location		No mention of user involvement in design and evaluation	
	[21]	48		No; borderline personality disorder also included		Mood Zoom smartphone questionnaire		No mention of user involvement in design and evaluation	
	[22]	118		Yes		Personal Life-Chart App: electronic diary–based smartphone app		No mention of user involvement in design	
	[23]	28		Yes		MONARCA^a^ system: combination of passive and active self-monitoring smartphone app amended to measure voice feature		No mention of user involvement in design	
**Low user involvement (n=1)**									
	[24]	76^b^		No; psychosis also included		Ginger.io: smartphone-based mental health tracking app		Users involved in evaluation stage	
**Medium user involvement (n=4)**									
	[25]	N/A^c^; this is a protocol		No; other severe mood disorders		E-care at home: tablet-based self-monitoring tool		Users involved prototype design and evaluation stage with iteration	
	[26,27]	7		Yes		MoodRhythm: smartphone app that can track social rhythms		Users involved in prototype design and evaluation stage with iteration	
	[28-30]	42 (all papers combined)		Yes		MONARCA system: combination of passive and active self- monitoring smartphone app		Users involved in prototype development and evaluation stage with iteration	
	[31-33]	303 (all papers combined)		Yes		OpenSIMPLE: smartphone-based psychoeducation program		Users involved in prototype development and evaluation stages with iteration	
**High user involvement (n=2)**									
	[34]	59^b^		No; other serious mental illnesses also included		QoL^d^-ME: smartphone-based, personalized QoL assessment app		Users were involved in concept generation and ideation stage, prototype design and deployment stage and evaluation stage with iteration with a goal to empower patient decision-making	
	[35,36]	25^b^		No; also included posttraumatic stress disorder		SPIRIT^e^ App: smartphone self-monitoring app that allows patients to undertake modules and complete questionnaires for mental health assessment		Users were involved in concept generation and ideation stage, prototype design, and deployment stage and evaluation stage with iteration with a goal to empower patient decision-making	

^a^MONARCA: Monitoring, Treatment and prediction of bipolar disorder episodes.

^b^Unclear how many of the participants had a diagnosis specifically of bipolar disorder.

^c^N/A: not applicable; the paper mentioned no user involvement.

^d^QoL: quality of life.

^e^SPIRIT: Study to Promote Innovation in Rural Integrated Telepsychiatry.

**Table 2:** Further summary of included studies including further description of methods used, model of user involvement and further description of user-involvement method.

Characteristics and reference			Further description of methods used		Model of user involvement		Further description of user-involvement model
**No user involvement mentioned (n=4)**							
	[20]	N/A^a^		N/A		N/A	
	[21]	N/A		N/A		N/A	
	[22]	N/A		N/A		N/A	
	[23]	N/A		N/A		N/A	
**Low user involvement (n=1)**							
	[24]	Users completed nonstandardized measures for satisfaction and perceived effect on clinical care		None mentioned		N/A	
**Medium user involvement (n=4)**							
	[25]	Prototype design and deployment stage: 3 rounds of interviews with 8 users where interactive demo materials and screenshots were provided as stimuli and feedback was used to iterate design Evaluation stage: Credibility and Expectancy Questionnaire, SUS,^b^ and Client Satisfaction Questionnaire administered to measure system usability, user experiences and client satisfaction.		Cocreation approach		Aim of the approach was to create a product that would be usable for the specific target population and move away from traditional rigid *waterfall* methods, which only have a single round of assessment or iteration. This approach affected the tool by uncovering usability requirements, which were implemented.	
	[26,27]	Prototype design and deployment stage: participants used the app and shared feedback, design insights and suggestions for improvement at least once a week. Wireframes were sent back to participants, which incorporated this feedback where further feedback was given Evaluation stage: poststudy usability scale using SUS		Participatory design process		During the design process, participants used the MoodRhythm app in their daily lives and shared their feedback, design insights, and suggestions for improvements to the app. This process allowed participants to provide feedback on an ongoing basis during the design process and helped to identify and address concerns that users might have regarding these technologies, ensuring the app was effective for daily use.	
	[28-30]	Prototype design and deployment stage: 3-hour workshops were held for design and iterative prototyping where feedback was incorporated into design Evaluation stage: SUS was administered in a field trial. A nonstandardized questionnaire for usefulness and perceived usefulness was also developed and administered.		Patient-Clinician Designer Framework using principles of user-centered design		Through this design process, users were “involved” in making decisions regarding system features using collaborative design workshops. The design of the MONARCA^c^ system uses a mobile phone app as the main component.	
	[31-33]	Prototype design and deployment stage: users were involved in focus groups, interviews, and surveys with research teams. Unclear how findings were used to iterate the prototype Evaluation stage: engagement was calculated based on weekly percentage of completed tasks. Usability was calculated using the SUS and satisfaction and perceived helpfulness using Likert scales.		User-centered design		Using the user-centered design approach, suggestions were incorporated based on feedback from the users during the feasibility study as well as modifications to adapt the platform for an open study. Several features were added to OpenSIMPLE using this approach	
**High user involvement (n=2)**							
	[34]	Concept generation and ideation stage: 10 participants were to share their experiences with smart devices, apps and QoL^d^ questionnaires and to ideate regarding QoL-ME in a focus group Prototype design and deployment: paper sketches (wireframes) were presented and were gradually refined, expanded, and made to function where a first prototype was developed. 25 participants were involved in this stage Evaluation stage: prototype was subjected to usability testing and systematically assessed using the SUS with a total of 25 participants. Goal to empower patient decision-making: no information was found in relation to this		Cocreation approach		The QoL-ME was cocreatively developed in an iterative development process with groups of people with severe mental health. The process consisted of 6 iterations divided over 3 stages: brainstorming stage, design stage, and usability stage. The development process was described as fitting in the framework of participatory design. Feedback was used to make several changes to QoL-ME	
	[35,36]	Concept generation and ideation stage: 1 focus group was run with users to propose the SPIRIT^e^ app and a second focus group was run to refine SPIRIT app concept Prototype design and deployment stage: focus groups were run with user to elicit feedback on storyboard and prototype and prototypes were refined based on feedback Evaluation stage: developed a usability testing framework, which was conducted with 5 participants where feedback was incorporated into the app Goal to empower patient decision-making and creating empathy: the study had an advisory group called CAB,^f^ which consisted of 8 “consumers” and “consumer advocates” who met monthly to advise the SPIRIT scientific team on all aspects of trial design and conduct, which was resulted in changes to the app and study		Human-centered design process, participatory design process, and Principle of Digital Development		Target users and domain experts were engaged in a participatory design process throughout development via focus groups and usability testing with national consumer advocacy groups and providers and patients in rural clinics. The process also adhered to the principles of digital development which includes the following: design with the user; understand the existing ecosystem; design for scale; build for sustainability; be data driven; use open standards, open data, open source, and open innovation; reuse and improve; address privacy and security; be collaborative	

^a^N/A: not applicable; the paper mentioned no user involvement.

^b^SUS: System Usability Scale.

^c^MONARCA: Monitoring, Treatment and prediction of bipolar disorder episodes.

^d^QoL: quality of life.

^e^SPIRIT: Study to Promote Innovation in Rural Integrated Telepsychiatry.

^f^CAB: Consumer Advisory Board.

### Expert Panel Review

As per the third question, the findings of this paper have been discussed within a group of individuals who offer academic and clinical expertise in this area as well as an individual with lived experience of BD who also has a good level of experience and participation in PPI. On the basis of these discussions, we recommend the following:

1. Involve users in all stages of design and evaluation, including concept generation and ideation, prototype design and deployment, and evaluation stages with the goal of creating user empathy and empowerment. This process should have an adequate number of participants to welcome diversity in thought. Equal representation is also a crucial consideration that needs to be considered when recruiting users.
2. Ensure early involvement as this will be cost-effective in the long run (avoid redesign and problems with use and implementation in the later stages).
3. Combine principles of PPI and HCI to not only have users to assist in designing technology but also in designing and running research (eg, users cofacilitating design workshops) and use end-to-end user involvement.
4. For academic and industry sectors to establish better mechanisms to access target user groups with lived experience of mental health issues, for example, by building relationships with existing patient-directed organizations such as charities and patient-led community groups.
5. Increase awareness of HCI and design communities in PPI principles and practices and increase awareness of PPI community in HCI and design methods or skills.
6. Encourage use and mixing of formal scientific or design methods with informal experiential and empathic practices to capture richness in understanding the dynamic requirements of technology users, which are cognizant of use in context.
7. Keep the user informed at all stages of the process, including final outcomes, future use, and next steps, which are often forgotten about.

## Discussion

### Principal Findings

This paper presents the current landscape regarding user-led design and evaluation of self-monitoring apps for BD. This was investigated via 3 specific questions: (1) are users involved in the design and evaluation of technology? (2) if so, how does this happen? and (3) what are the best practice *ingredients* regarding the design of mental health technology?

For the first question, a total of 17 papers were included in this review, which resulted in the evaluation of 11 novel smartphone apps for self-monitoring of BD. In total, 6 of these papers have been grouped together as they have been published with respect to the same smartphone app, which is highlighted in the Tables 1 and 2. Regarding the first question, the results from this review indicate that users are being involved in design and evaluation, but this is highly variable in terms of level of involvement. In total, 4 self-monitoring apps (n=4 papers) were classified as having no mention of user involvement in design, 1 self-monitoring app (n=1 paper) was classified as having low user involvement, 4 self-monitoring apps (n=9 papers) were classified as having medium user involvement and 2 self-monitoring apps (n=3 papers) were classified as having high user involvement.

With respect to the second question, there is variability in the models of user involvement in design and evaluation, where the following have been described: agile development process, cocreation approach, participatory design, patient-clinician designer framework, user- and human-centered design, and principles of digital development. The key characteristics of the models are described in Textbox 2. The standout method mentioned was the *agile development process*, which is a software development process aimed at producing outcomes fast in relation to market constraints and the ability to accommodate changes during the software development cycle [37]. The use of mental health technology is sparse [38], and there are questions as to how a model aimed at quickly meeting market constraints considers the users’ voice and needs during the process of designing mental technology, which sheds light on why it was ranked low regarding involvement as it only considered users in the evaluation stage. Finally, this large amount of variability sheds further light on the need for quality guidelines in the reporting of user-involved development of mental health technologies.

**Key characteristics of user involvement.**

**Agile development process**

- An iterative approach to project management which is aimed at product fast outcomes in relation to market constraints and the ability to accommodate changes during the software development cycle

**Cocreation approach**

- A process which is aimed at creating “with” users and stakeholders to ensure results meet their needs and are usable

**Patient-clinician designer framework**

- A process which uses the key principles of user-centered design to be applied in the context of mental health. A framework which aims to involve patients and clinicians in the process of design through collaborative design workshops and iterative prototyping

**Human-centered design**

- A process which is based on designing based on characteristics and intricacies of human psychology and perception which is considered to carry out a deeper analysis that user-centered design

**Principles of digital development**

- A process which focuses on the following 9 principles during digital development:
  Design with the user
  Understand the ecosystem
  Design for scale
  Build for sustainability
  Be data driven
  Use open data, open standards, open source, and open innovation
  Reuse and improve
  Address privacy and security
  Be collaborative

The descriptive section of Textbox 2, which describes how models of involvement were implemented as outlined in the selected papers, also uncovers pertinent findings to the second question. It was found that papers that were classified as having high user involvement displayed an increased level of detail on how they implemented their chosen user involvement model. In addition, those papers that were classed as high user involvement not only described their methods as mainly participatory design, but also described a combination of methods such as cocreation, participatory design, and human-centered design. This highlights some early suggestions that it is the combination of methods that could be the driving force of ideal user involvement, which underpins points 5 and 6 of our recommendations of best practice, as outlined in the *Results* section. These points indicate that there should be a mix of methods to capture the unique and dynamic requirements of mental health technology users and that there should be an increased awareness of these methods in both the HCI and PPI design communities.

Focus groups were the method of choice during the concept generation and ideation stages. For the prototype design and deployment stage, the following methods were shown to have been used: focus groups and workshops, sharing of wireframes, and interviews. For evaluation stages, there was a combination of the use of standardized and unstandardized questionnaires to measure factors such as usability, satisfaction, and usefulness of the smartphone app. For a few studies, completing tasks using the app was also a method used for evaluation. Only 1 study that met the high user involvement criteria had explicit reference to empower patient decisions and create empathy by having a patient advisory board whose role was to advise the scientific team of all aspects of the study and smartphone app. Some studies [26,27] had particularly low participant numbers (ranging from 1 to 7 users), and there are questions as to whether such small samples can adequately capture users’ needs or wants and whether this constitutes a user-focused approach. The aims of these methods are to represent diversity in this voice and capture both an implicit and explicit understanding of users, tasks, environments, and interactions so that technology can be designed better, and it can be argued that such small samples cannot provide the richness of understanding needed for this. The real lived experience of the condition cannot be understood and adequately covered with such a small sample size. That is not to say that a large number of participants capture this adequately either as there is a pool of studies [21,22,24] with a large group of participants that do not adequately describe how the findings were used to iterate the technology. It is both the number of users and the level of engagement that constitutes an appropriate user-centered methodology. In summary, this research provides evidence that despite the presence of recommended standards for the involvement of the user in the process of design and evaluation of mental health technology specifically for BD, there is large variability in whether the user is involved, how they are involved, and to what extent there is genuine empowerment in the voice of the user, which is the aim of design approaches involved in mental health technology.

### Limitations

The limitations of this study may also contribute toward the lack of user involvement mentioned in this paper. This paper reflects the current practices of user involvement to the extent to which authors made this explicitly available in the chosen literature. It could be the case that not all authors disclosed the process of design in the paper for a variety of reasons. With strict word limits in the case of often complex papers in the field of mental health technology, authors may have decided to focus on other parts of the technology, such as results, and omit the design and development of the technology. Likewise, the process of design could be described in other papers, which may not have been included in this review. We did not contact authors to check if there was additional literature on user design or interview authors regarding user design in the development of technology, but such practices might produce a more comprehensive review of user design practices in the future. It is also worth noting as a limitation that only research-led app development projects were assessed and there may be innovations in commercial and nonprofit developments that have not been considered in this paper as only published literature was considered. Finally, there may be limitations around the recommendations of best practice provided by the group of experts, and future studies should consider more structured tools such as the Delphi method.

### Comparison With Previous Work

A question for future investigation is why is this variability present? Previous studies have considered this, where it was concluded that there is a lack of parity when involving those with mental health issues in design compared with those with physical health issues [6] and more specifically for BD it was found that only a small proportion of studies for technology included involving the user in a recent review [7], despite the benefits of involvement [9]. The first step to consider is the inherent logistical and ethical issues that arise when working with those with severe mental health issues [39]. In terms of studies that indicated no or low user involvement, 1 potential reason for this may be accessibility to a suitable user group. Academic institutions are often closely linked to health care settings with formal protocols and regulations that allow accessibility to mental health care settings and patients. However, there are drawbacks to this, including only being able to access the same user representatives, which is likely to create inherent bias. In contrast, research conducted in industry settings is likely to not have this type of working relationship, largely because of conflicts of interest, making it more difficult to access the right user group. Academic, clinical, and industrial settings need to work collaboratively to establish mechanisms to enable technology development and the contribution of appropriate users to be accessible, inclusive, and representative.

From a more systemic perspective, this intersection between PPI and HCI may shed more light on the variability of user involvement both in this study and more generally. Both approaches combined describe the umbrella of the types of user involvement methods described in this review. However, both approaches individually have tensions that need to be considered, which are likely to impact the application of these methods. PPI approaches have limitations in that they are currently not versed in considering design within the tensions of cost and regulation, as PPI processes are often the result of existing government funding for research, rather than commercial and industrial funding, which is more typical of technology development. Tensions arise because of the differences in time management and resource allocation depending on the funding source, and there are questions regarding the adaptability of PPI practices for this. These practices tend to be focused on the clinical context and clinical task and are not versed in considering more subjective, hard-to-measure, and tacit aspects when designing technology such as user experience and everyday life practices, which are facets removed from direct clinical care. PPI approaches tend to traditionally linear, static approaches that do not evolve or iterate owing to new information, which is not suitable when designing technology where iteration is a requirement. This is because the origins of PPI do not stem from design or scientific disciplines, such as HCI, where the elicitation of need-finding is not just limited to the anticipated as it is in PPI but also unanticipated or implicit in nature [40]. For example, when reviewing user preferences on data visualization for remote monitoring technology, BD was touched upon, and it was found that the state of readiness and state of health as well as data literacy and familiarity with technology are all factors when considering user engagement with remote monitoring technology [40]. The consideration of factors, such as state of readiness may not be readily captured by PPI.

For HCI approaches, unlike PPI processes, there is a lack of use of these formal methods in the context of mental health service and technology design. Therefore, there is little evidence of their effectiveness [17]. PPI processes based on and in health care provision and improvement work are often conducted by professionals who have a skillset targeted toward working and engaging with those with mental health issues, whereas this cannot be said in the field of HCI where the training background is largely different with little or no experience in mental health. It is unclear whether this is a benefit of technology-based approaches as it has the potential, if planned and implemented well, to remove the power dynamics and hierarchy by not having a clinician taking lead which can sometimes negatively impact meaningful PPI contributions. Alternatively, it may be that if not designed with empathy and in conjunction with the advice from clinical persons or those with lived experience, HCI approaches could be a hindrance and a barrier to disclosure and engagement if the nonclinical professionals do not have the skillset to meaningfully engage those with mental health issues. This research highlights the need to upskill both communities to be better equipped, and it is important for future research should aim to explore this. Conclusively, user-focused approaches can provide a framework for PPI to embed participatory activities within the iterative, fast-paced development process of mental health technology development. Likewise, PPI has developed core standards around establishing an equal relationship between users and researchers, which can lend itself well in HCI approaches where this is not necessarily present.

### Conclusions

In conclusion, this research provides evidence that despite recommendations on the involvement of users in the process of mental health technology design and evaluation, in this case, specifically for BD, there is large variability in whether the user is involved, how they are involved, and the extent to which there is authentic empowerment of the user’s voice. The tensions among the design approaches used in PPI and HCI may shed some light on why there is variability in user involvement. Currently, both design approaches work independently; however, future practices should aim to work together and encourage awareness and mixing of methods. The findings of this research have been reviewed by an expert panel, including an individual with lived experience of BD, and recommendations were made for the design communities to establish better mechanisms for awareness, mixing of methods, and increased user involvement.

## Abbreviations

BD: bipolar disorder
HCI: human-computer interaction
PPI: patient and public involvement
UCD: user-centered design
